# Technical Tips and Tricks after 10 Years of HyFoSy for Tubal Patency Testing

**DOI:** 10.3390/jcm11195946

**Published:** 2022-10-08

**Authors:** Roxana-Elena Bohîlțea, Bianca-Margareta Mihai, Cătălina-Diana Stănică, Consuela-Mădălina Gheorghe, Costin Berceanu, Vlad Dima, Alexia-Teodora Bohîlțea, Smaranda Neagu, Radu Vlădăreanu

**Affiliations:** 1 Department of Obstetrics and Gynaecology, ‘Carol Davila’ University of Medicine and Pharmacy, 020021 Bucharest, Romania; 2 Department of Obstetrics, Gynaecology and Neonatology, ‘Filantropia’ Clinical Hospital, 011132 Bucharest, Romania; 3 Department of Marketing and Medical Technology, ‘Carol Davila’ University of Medicine and Pharmacy Bucharest, 050474 Bucharest, Romania; 4 Department of Obstetrics and Gynaecology, University of Medicine and Pharmacy of Craiova, 200349 Craiova, Romania; 5 EHL Swiss Hospitality Management School, 1000 Lausanne, Switzerland; 6 Heinrich-Heine-Universität Düsseldorf, Humanmedizin, Universitätsstraße 1, 40225 Düsseldorf, Germany; 7 Department of Obstetrics and Gynaecology, Elias University Emergency Hospital, 011461 Bucharest, Romania

**Keywords:** HyFoSy, hysterosalpingo-foam sonography, tubal patency, pain, infertility

## Abstract

Background: Hysterosalpingo-foam sonography (HyFoSy) has gained popularity in the last decades, as it represents a feasible, well-tolerated, and minimally invasive method of evaluation of tubal patency in cases of infertility. The purpose of this study was to communicate the technical tips and tricks based on our experience in performing HyFoSy, with the aim to improve the feasibility, to reduce the pain, and to evaluate pregnancy-obtaining rate after procedure. Methods: Our observational study includes 672 patients from infertile couples who underwent HyFoSy for tubal patency evaluation. During HyFoSy, tubal pathway and patency as well as the level of pain were evaluated. A telephonic questionnaire was conducted in order to assess the pregnancy obtaining rate in the first 3 months and more than 3 months after the procedure. Results: The median age in our group was 33.5 years. Most of our patients (61.16%) underwent HyFoSy in the 8–10 days of the menstrual cycle. Tubal patency was present bilaterally in 86% cases, unilaterally in 11% of patients, and was absent in 3% of cases; 75% of patients related absent or tolerable pain, 17% described HyFoSy as a painful procedure, and 8% experienced extreme pain. After HyFoSy, pregnancy was naturally obtained in 10.86% of cases within the first 3 months after HyFoSy. Conclusions: HyFoSy represents a useful, easy to use, and painless tool in female infertility evaluation and should be considered as a complementary method of the transvaginal ultrasonography, completing the genital tract imaging with information about the hidden part of the standard examination: tubal patency. HyFoSy provides information about patency, caliber regularity, pathway, and occlusion location of the fallopian tubes; therefore, it should be introduced along with transvaginal ultrasound as a first-line infertility exploration method.

## 1. Introduction

Tubal patency represents an important step in infertility investigation, as tubal anomalies have been identified in 30–40% of women affected by infertility [1]. The current tubal permeability evaluation procedures include hysterosalpingography, hysterosalpingo-contrast sonography, as well as diagnostic laparoscopy with chromopertubation [2]. Hysterosalpingography represents an investigation done under fluoroscopic guidance, presenting 53% sensitivity and 87% specificity for diagnosing tubal pathologies; therefore, it is able to infirm tubal occlusion but is not the best procedure for diagnosing discrete pathologies such as peritubal adhesions [3]. The main disadvantages consist of the need of a radiology department, the possible allergic reactions to iodinated contrast substance, and the patient’s exposure to radiation [4]. Diagnostic laparoscopy with chromopertubation testing [5], along with the pelvic evaluation, permits surgical treatment of potentially found pathologies causing subfertility or infertility, the most frequent being endometriotic lesions [6]; the procedure is performed as inpatient, and it is minimally invasive but presents subsequent surgical and anesthetic risks, an elevated cost, and long waiting time for patients [7].

Hysterosalpingo-contrast sonography (HyCoSy) has gained popularity among the examination methods of tubal patency, as the patient’s exposure to radiation is avoided, and it is less invasive but with sensitivity and specificity comparable to those of hysterosalpingography [3]. Many contrast agents have been used in time since HyCoSy was first described in 1984 by Richman et al. [8], who used Hyskon, a diluted solution of dextran. Two years later, Echovist-200, a D-galactose microparticles solution used in Europe for cardiac catheterization, was utilized as contrast agent in HyCoSy and compared with hysterosalpingography and laparoscopy, having impressive results. The disadvantage of Echovist−200 was a short duration of visibility through the fallopian tubes, necessitating an experienced sonographer to perform the tubal investigation [9].

Saline-infused HyCoSy was firstly used to investigate infertility, which had remarkable results on detecting uterine pathology, but tubal patency was difficult to assess [10].

In 2007, a gel for hysterosonography (ExEm Gel), gel instillation was introduced as an alternative for saline infusion [11]. In 2011, a new technique, HyFoSy, was reported, using foam-based gel for ultrasound tubal patency testing [12]. ExEm gel foam is registered and approved by the European Conformity for tubal permeability evaluation [13,14].

Second-generation contrast agents using phospholipid-coated fluorene bubbles (SonoVue, Definity) had a longer duration of ultrasonographic visualization but presented rare adverse reactions such as anaphylaxis, and although approved by the Food and Drug Administration (FDA) in the United States for intravenous use, they are not recommended for intrauterine use [15].

Other invasive methods of tubal patency exploration include office hysteroscopy with selective perturbation, described by Török and Major [16], and transvaginal salpingoscopy, reported by Gordts et al. [17]. A recent systematic review published by Vitale et al. in 2020 [18], which evaluated the diagnostic accuracy of hysteroscopic chromopertubation used in tubal patency exploration, concluded that although encouraging, the diagnostic efficacy of hysteroscopic techniques regarding tubal occlusion must be confirmed with the help of larger studies.

While the most frequent cause of tubal disfunction is represented by pelvic inflammatory disease, there is one factor that has not been enough discussed, namely the tubal microbiota, which could play a role in maintaining the reproductive function. One recent paper was published in 2022, exposing the different methods that could be helpful in evaluating the tubal microbiota. A promising method could be atraumatic hysteroscopic sampling, but it requires larger studies evaluating its role in order to evaluate the association between tubal microbiota and female infertility [19]. In addition, hysteroscopy as an interventional procedure can be used in certain cases of infertility such as dysmorphic uteri, in which hysteroscopic metroplasty represents a minimally invasive approach, improving fertility [20].

Regardless of the method, the result of tubal patency testing, in case of a negative result demonstrating the presence of tubal patency, should be accepted (if properly used), while a positive result (tubal occlusion) is not assuring due to the possibility of prolonged contraction, blockage of the tip of the catheter, etc.

The aim of this study was to evaluate the technical success rate of HyFoSy use in diagnosis of tubal causes of infertility and the pain perception during this procedure as well as another debated subject in the literature and the improvement of pregnancy occurrence after the procedure. Therefore, we propose, after 10 years of the first reported results in tubal patency exploration and after a vast personal experience, tips and tricks for the best technical results.

## 2. Materials and Methods

In this observational study, we included 672 women who presented consecutively for infertility assessment in our private medical unit, the Maternal–Fetal Medicine Department of Medlife Hyperclinic, during January 2013–May 2021. Tubal patency evaluation was performed using HyFoSy as a complementary exploration method after clinical examination and routine transvaginal ultrasonography.

Infertility was diagnosed on standard criteria: absence of conception after 12 months of regular unprotected sexual intercourse in women before the age of 35, respectively, and more than 6 months of unprotected regular intercourse for women aged 35 or older [21].

**Figure 1:**
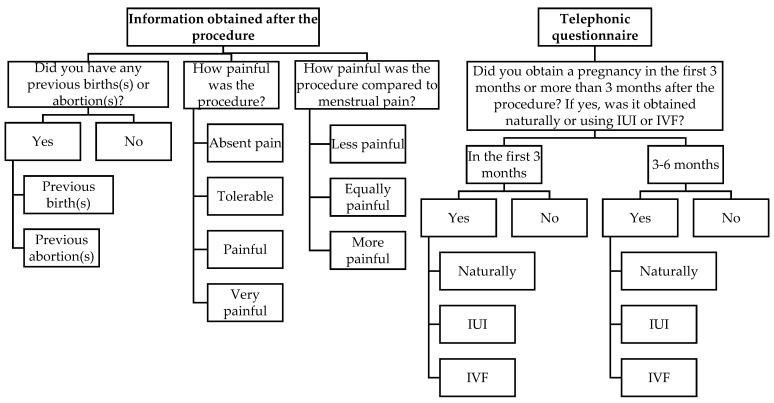
The information obtained after the procedure and the telephonic questionnaire applied to every patient in our study (IUI, intrauterine insemination; IVF, in vitro fertilization).

The medical report of each patient was screened for information, and a telephonic questionnaire was conducted, including a standardized quiz with multiple answers from which the patient must choose one. The patients consented to participate in our study database, with the procedure results, and to answer a future telephonic questionnaire 6 months after the procedure. We respected the patients’ personal data privacy. The questions from the HyFoSy procedure and from the telephonic quiz are summarized in Figure 1. The information about pain perception was collected at the end of the procedure; the telephonic questionnaire had the main aim to obtain information about pregnancy occurrence in the first 3 months or between 3–6 months after HyFoSy. Regarding pain perception of the HyFoSy procedure, we used the numeric rating scale [22] from 0 to 10, and we grouped the results into four categories: absent pain (0), tolerable (1–3), painful (4–6), and very painful (7–10).

Preparing the patient for HyFoSy includes the presence of a signed informed consent, microbiological genital testing results, antispastic drugs administered before the procedure (1 tablet of 80 mg drotaverine hydrochloride in the evening and 1 tablet in the morning before the procedure), medicinal charcoal administration, and a single dose of 100 mg doxycycline taken 2 h before the investigation. In the last menstrual cycle before the procedure, we always test the vaginal flora; cervical pathogenic germs, excluding *Chlamydia trachomatis*, *Mycoplasma hominis*, and *Ureaplasma urealyticum* infections; high-grade cervical anomalies; and, of course, pregnancy. The procedure is scheduled in the proliferative phase of the menstrual cycle.

**Figure 2:**
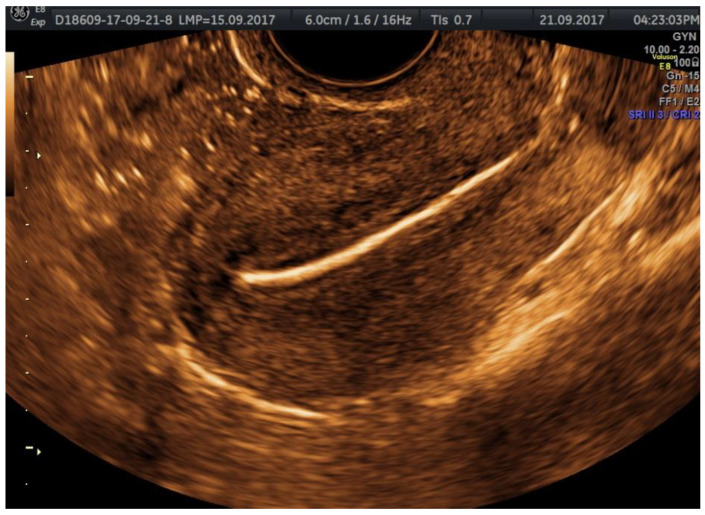
2D HyFoSy, sepia mode: the gradual penetration of the gel allows the visualization of the uterine endocavitary contour; the endometrium is optimally examined before the instillation, appearing thin, hypoechoic, and homogeneous in the early proliferative phase.

**Figure 3:**
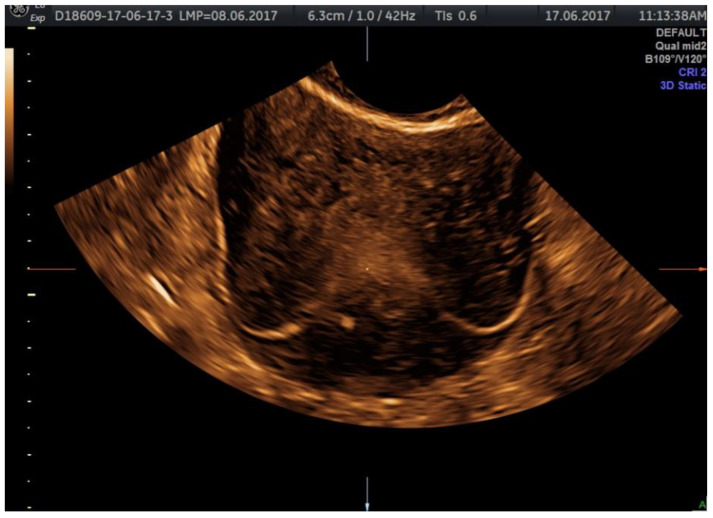
3D-ultrasonography static mode HyFoSy: the intramyometrial segment of the fallopian tubes.

**Figure 4:**
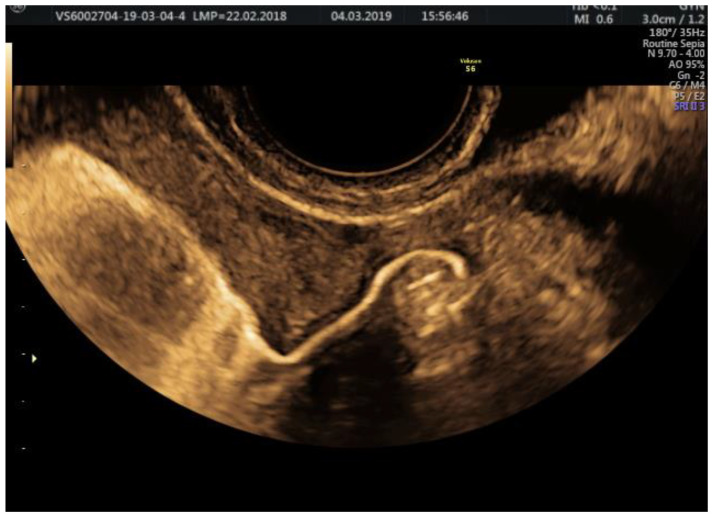
2D HyFoSy, sepia mode: left fallopian tube, patent with straight regular pathway.

**Figure 5:**
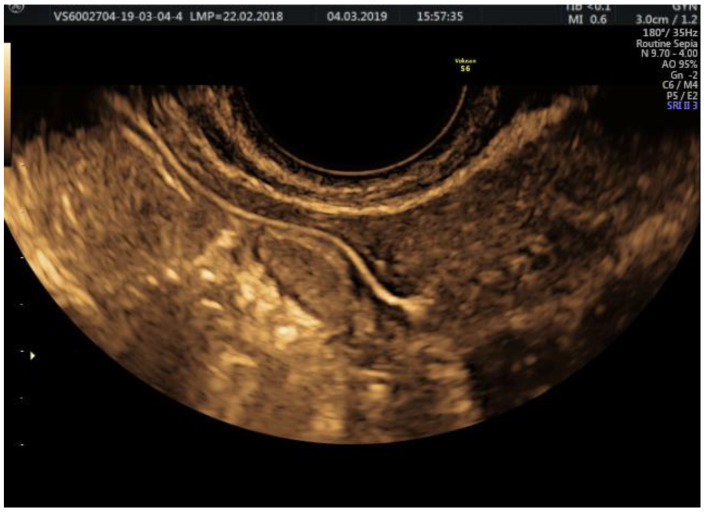
2D HyFoSy, sepia mode: right fallopian tube, patent with straight regular pathway.

The examination kit contains a 10 mL syringe with gel composed of hydroxyethylcellulose and glycerol, a syringe with purified water, an empty syringe and a coupling device, as well as a catheter. Autostatic valves (a disposable plastic speculum) and transvaginal ultrasonography equipment without contrast software are also required; we performed the procedure using a Voluson E8, equipped with a Ge Rab4-8-D Ultrasound Probe, mainly using 2D or 3D sepia mode (Figure 2, Figure 3, Figure 4 and Figure 5).

The data collected did not contain personal information, and Ethics Committee approval of the Medlife Hyperclinic was required and obtained (protocol code 30/18.10.2016). The procedures we performed respected the ethical standards in the Helsinki Declaration of 1975, as revised in 2000, as well as the national law.

## 3. Results

Our group included 672 infertile women undergoing HyFoSy in our private medical clinic. The minimum age was 20 years, the maximum age was 46 years, and the median age was 33.5 years. The age group with the most frequent infertility exploration was 31–35 years. Additionally, 50% of the explored women were under the age of 33 years.

**Figure 6:**
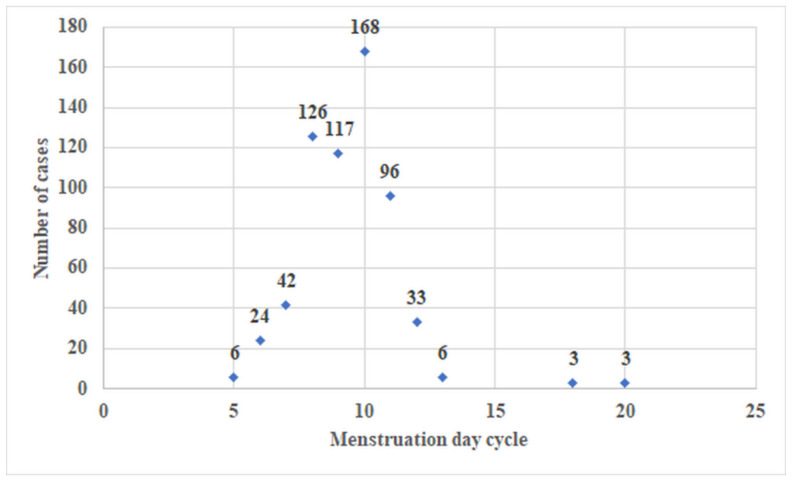
The distribution of cases on menstrual cycle day in which the HyFoSy was performed.

The patients were explored in the proliferative phase of the menstrual cycle, with most of the procedures (411, 61.16%) being realized between the days 8–10 of the menstrual cycle (Figure 6).

During HyFoSy-enhanced exploration, the uterine cavity contour and the path and patency of the fallopian tubes were evaluated. The great majority of our cases (59%) presented a regular path of the fallopian tube, while almost one-third (30%) presented a tortuous path, 10% were filiform, and 1% had an irregular path. The tubal patency in our infertility cases was present bilaterally in 86% of cases, while in 11% of cases, the contrast substance revealed unilateral permeability, and in 3% of cases, the permeability was absent bilaterally.

Regarding the endocavitary pathology, the most frequent deforming cause was represented by endometrial polyps (93 cases), and another nine cases of intracavitary leiomyomas, three cases of both polyps and intracavitary leiomyomas, and twelve cases of Mullerian anomalies were diagnosed. According to the ESRE classification [23,24], we found three cases of septate uterus (class U2) and nine cases of bicornuate uterus (class U3). Regular uterine cavity (class U0) was found in 525 patients. All polyps were removed by hysteroscopy regardless of their size and absence of symptomatology.

The success of HyFoSy depends on the success of the cervical cannulation and the gel progression through the uterine tubes. In our study, we had 102 cases (15.4%) of difficult penetration of the gel, 75 cases (11.1%) with difficult cannulation, and procedure failure in 4 cases (0.59%), mainly due to cervical stenosis that could not be overcome in outpatient conditions.

Within the group, 47% of our patients were diagnosed prior to the procedure with secondary sterility (Table 1). According to our telephonic questionnaire, after HyFoSy, pregnancy was obtained in 37% of cases: 19% of patients obtained pregnancy within the first 3 months after HyFoSy and 18% between 3 and 6 months. In the first 3 months, 57% of pregnancies were obtained naturally (73 cases), 11% were obtained using IUI (14 cases), and 32% were obtained using IVF (41 cases).

Pain during the procedure was evaluated using the pain scale (Table 2) and in relation to menstrual pain (Table 2). Regarding pain, 75% of patients related absent or tolerable pain, 17% described HyFoSy as a painful procedure, and 8% experienced extreme pain. Compared to menstrual pain, 363 (54%) of patients considered the procedure less painful than menstrual pain, 101 (15%) patients found HyFoSy as painful as menstrual pain, and 208 (31%) patients experienced more pain during HyFoSy than during menstruation.

## 4. Discussion

This study is the first describing tips and tricks to improve and standardize the HyFoSy technique in order to obtain excellent examination results. The use of gel foam in hysterosalpingo-contrast sonography offers a high diagnosis accuracy of tubal patency with the minimum discomfort experienced by the patient, a fact sustained by Van Schoubroeck et al. [14] in an article published in 2013 that reported a 100% diagnosis agreement between laparoscopic chromopertubation and HyFoSy.

Concerning feasibility, Engels et al. [25] related eight cases (0.87%) in which the procedure failed due to impossibility of introducing the intracervical catheter. We encountered the same issue in four cases (0.59%), with only one case of cervical stenosis. The solution to difficult canulation is the use of progressive cervical dilators (Hegar) from 1 to 3 mm, avoiding as much as possible the application of cervical forceps and traction, respectively, and vasovagal reactions; when necessary, we apply local anesthetic.

Based upon our experience following our study, we managed to define tips and tricks that we consider helpful for all sonographists in order to achieve the best technical results.

### 4.1. Tips and Tricks

The main condition in order to obtain a successful result is represented by scheduling the procedure in the first or second day after menstrual bleeding cessation because the cervical canal is significantly easier to cannulate. In 15.4% of explored cases, after introducing the catheter, extracting the speculum, and introducing the transvaginal probe, at the beginning of the instillation, even if the catheter is appropriately placed in the cervix, the substance was introduced with difficulty. In almost all these cases, we found at least one tubal obstruction, and the pain was more intense, suggesting an increased pressure to the uterine cavity and to the fallopian tubes; opposite to this increased resistance, the abnormal lower resistance to the flow has the significance of substance reflux due to the accidental redraw of the catheter, with instillation and subsequent loss of the substance into the vagina.

We recommend the use of a wedge-shaped pad or of a gynecological table with an easy approach to the sonographic exploration, being able to ensure a gynecological position associated to the Trendelenburg position as close as possible to 45 degrees. This position favors the gut displacement towards the cranial direction and also repositioning the fallopian tubes as anatomically as possible, so they may be easily spotted.

The psychological factor is also an element, which should be considered. The patient must be assured that the procedure is not more painful than menstrual pain because fear causes spasm of the external cervical orifice, which could make the cannulation difficult. By explaining step by step the procedure to the patient, the cannulation could be facilitated [26].

The preprocedural preparation with medicinal charcoal and drotaverine hydrochloride is helpful in the tubal patency visualization in optimum conditions without a distended gut. Medicinal charcoal is used in reducing bloating [27], and its administration could be considered an innovative tool for increasing the accuracy of the ultrasound investigation by the lack of overlapping ultrasonographic images of the gas-distended gut. For the same reason, we use the wedge-shaped pad.

Always test the vaginal flora. This step is fundamental in infections prevention. In our group of more than 600 participants, there were no consecutive pelvic inflammatory disease or tubo-ovarian abscesses recorded. We take a supplementary protective measure by a single doxycycline dose administration and vulvo-vaginal disinfection with betadine.

We use a large-sized autostatic speculum to permit maneuvers. Autostatic valves (plastic speculum) are used to reduce the discomfort of the patients, but the main reason for use is the free movement of the sonographer, so he can use both hands for the cannulation of the cervix, a step that almost every time requires a cotton swab forceps, which has the role to push the gel instillation device in order to progress along the cervical canal.

**Figure 7:**
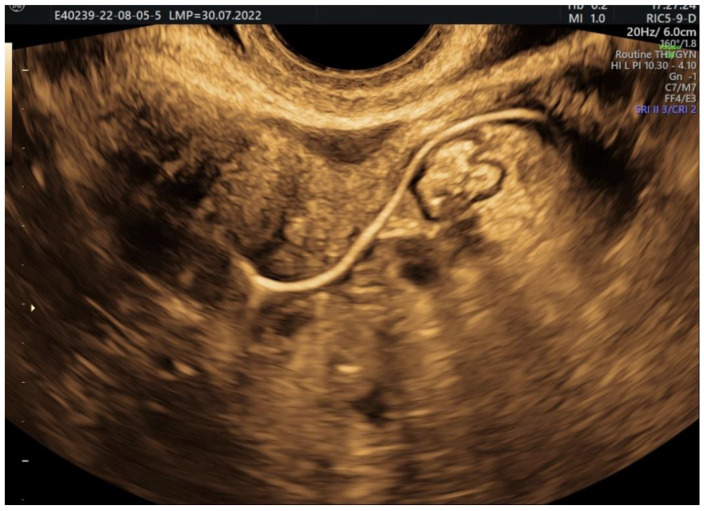
2D HyFoSy, sepia mode: left gel-opacified fallopian tube. Dynamic evaluation in which we can observe the same caliber of the tube from its intramyometrial portion up to its end, near the ovary; patent with straight, regular pathway.

**Figure 8:**
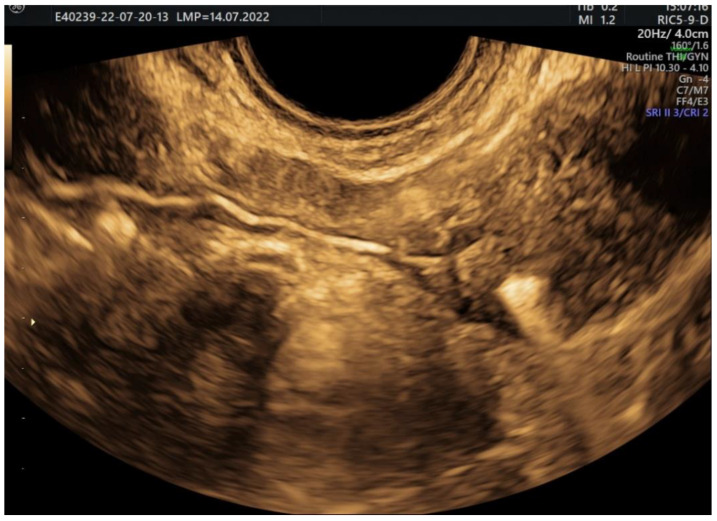
2D HyFoSy, sepia mode: right gel-opacified fallopian tube. Dynamic evaluation in which we can observe its regular caliber and sinusoid pathway.

We insist upon the slow release of the contrast substance, tracking the substance progression, and pausing the injection so that the substance would be quantitative enough for the visualization of both fallopian tubes (Figure 7 and Figure 8).

**Figure 9:**
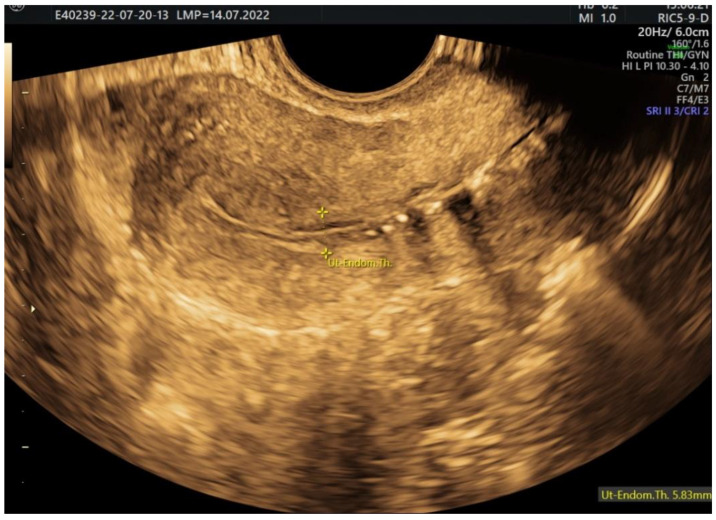
2D HyFoSy, sepia mode: initial measurement of the endometrium before the instillation of the contrast substance.

We measure the endometrium thickness before the injection of the substance (Figure 9). If there is anything that could be deforming the uterine cavity (e.g., polyp, myoma), it is optimally visible at the end of the procedure, when the uterine cavity is not distended due to the substance, so the focal lesions are highlighted by a fine layer of substance.

The tubal patency is confirmed by visualizing the contrast substance progression through the entire tubal pathway, its evacuation near the ovary, and finding the contrast substance at the end of the procedure in the pouch of Douglas or as a fine hyperechoic line near the uterus. When we speak about an irregular moniliform tubal pathway, we always think about genital tuberculosis-induced modifications, a pathology that is not eradicated in Romania and can be a tubal cause of infertility. In our experience, filiform tubes are potential causes of tubal ectopic pregnancies and moreover, subfertility.

**Figure 10:**
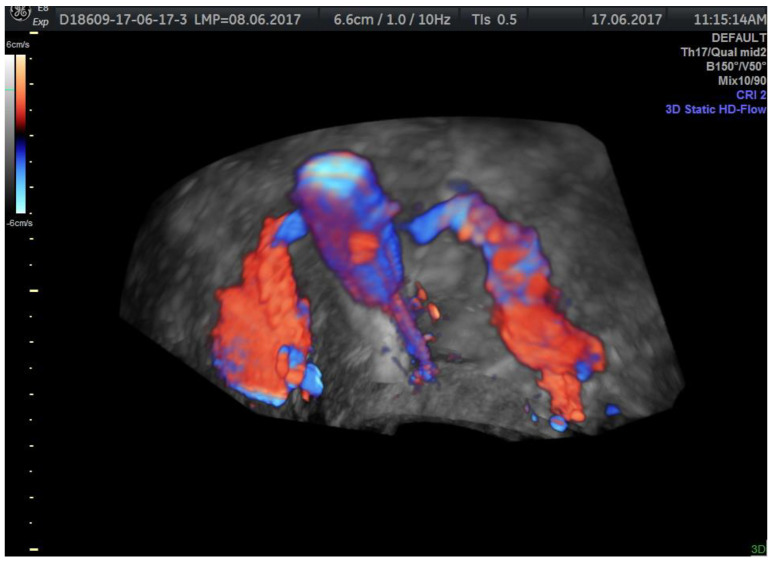
3D, HD-Flow color HyFoSy highlighting the contour of the endometrial cavity with vortex flow of the substance and the bilateral tubal passage to the pavilion level.

**Figure 11:**
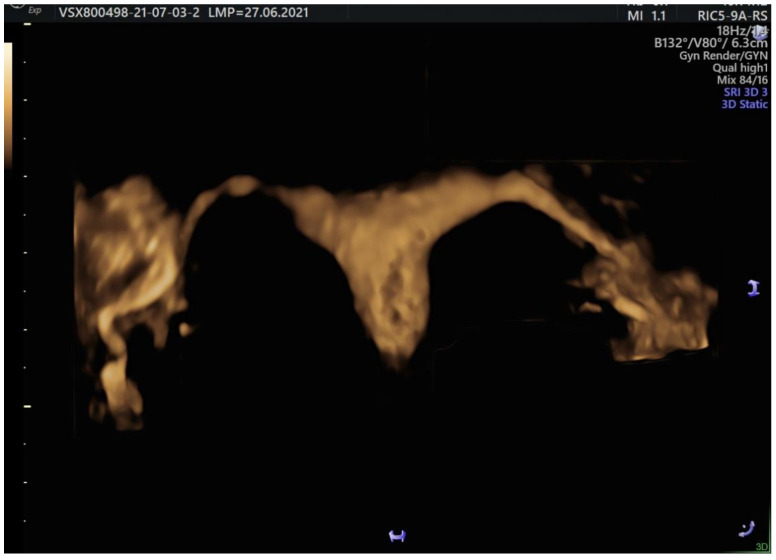
3D static HyFoSy with tubal patency and straight pathway of the fallopian tubes.

Using 3D mode on HD-Flow offers spectacular images (Figure 10 and Figure 11) without acquiring a real informational benefit. The advantage of HyFoSy relies upon the fact that the functional dynamics of the tubes are optimally evaluated by this method when used in sepia mode. Compared to hysterosalpingography under fluoroscopic guidance that captures successive frames of the substance progression and also to the saline solution elimination near the ovary appearing as a vortex flow on color Doppler, HyFoSy does not pass through the entire tubal pathway with high speed due to its high density; that is the reason why the tubal visualization is realized in real time simultaneously with the slow instillation of the substance through the catheter. In case of fast instillation, tubal distension is responsible for the increased pain level. If the instillation flow is too slow, the gel column does not progress through the tube and is not able to expose the whole length of the salpinx.

We compared our technical tips and tricks with the current literature and summarize the results in Table 3.

Regarding pain, a study published in 2014 [32] comparing pain scores during HyFoSy and hysterosalpingography concluded that patients undergo a lower pain during HyFoSy. An observational study realized in five Spanish centers, including 915 patients who underwent HyFoSy for infertility, by Engels et al., published in 2021 [25], evaluated the pain felt during HyFoSy using the visual analogue score (VAS) and ranging from 0 to 10, 0 being used in case of discomfort or pain absence, respectively, and 10 describing severe pain. The median VAS score in the study was 2; of the 915 patients, 62.2% and 35.8% described mild and moderate pain, respectively, and only 2% of patients experienced severe pain. In our study, 75% of patients related absent or tolerable pain, 17% described HyFoSy as a painful procedure, and 8% experienced extreme pain. Fallopian tubal obstruction is certainly associated with increased pain experienced during any tubal patency verification procedure even in office hysteroscopy [33].

Pregnancy occurrence in the first 3 months (19%) and more than 3 months after HyFoSy (18%) in our study was similar to the fertility rates in the literature: Emanuel et al. [13] related a 19% pregnancy occurrence rate within a median of 3 months after HyFoSy. Another study [34] published by Tanaka et al. in 2018 revealed a 30.9% pregnancy occurrence rate within the first 6 months using artificial reproductive technologies or that obtained spontaneously. A systematic review by Piccioni et al. published in 2020 [35] concluded that tubal flushing, including by HyFoSy, improved the embryo implantation rate and spontaneously obtaining pregnancy; thus, HyFoSy may also have a therapeutic role besides the diagnostic one. In the literature, there is a current opinion sustaining the idea that tubal flushing realized by HyFoSy is able to improve the chance of spontaneous pregnancy. The phenomenon was also observed after chromopertubation. Even if there are many studies in the literature sustaining the increase of the pregnancy occurrence rate after HyFoSy, our data does not support these results.

Regarding costs differences between available methods of tubal patency assessment, according to the FOAM study [36] published in 2018 in the Netherlands, the savings is about 100 euros per procedure for HyFoSy, which is detrimental for hysterography.

By using our technical recommendations, we eliminated the main elements that retrain sonographers from tubal patency evaluation, also described by Exacoustos et al. [37], namely tubal spasm, overlapping ultrasonographic images of the surrounding organs (ovaries, gut, etc.), and the manual capacities and experience of the examinator, and also perfected our technique in order to correctly visualize the tubes.

### 4.2. Strengths and Limitations

Firstly, our study’s major strength is represented by the fact that it is the first presenting technical tips and tricks in order to improve HyFoSy techniques and results. Secondly, we included a significant number of participants, 672 patients, which permitted an important experience acquisition, allowing us to perfect our techniques and further to define our technical tips and tricks. The primary limitation of our study is the fact that we did not include a control group for the pain during the procedure, as we did not have a significant number of participants consenting to a second tubal patency evaluation method in order to compare.

## 5. Conclusions

HyFoSy represents a useful, easy to use, and painless tool in female infertility evaluation and should be considered as a complementary method of the transvaginal ultrasonography, completing the genital tract imaging with information about the hidden part of the standard examination: tubal patency. HyFoSy provides information about patency, caliber regularity, pathway, and occlusion location of the fallopian tubes; therefore, it should be regarded along with transvaginal ultrasound as the first-line exploration of infertility.

HyFoSy is also a minimally invasive, painless, and dynamic procedure for tubal patency evaluation, with the advantage of a fast-learning level of use for sonographers while acquiring important information about the reproductive tract.

## Figures and Tables

**Table 1:** Results to the telephonic questionnaire regarding fertility.

	Number of Cases	Percentage (%)
Previous birth(s)	114	17
Previous abortion(s)	201	30
Absence of pregnancy after HyFoSy	426	63
Presence of pregnancy after HyFoSy	246	37
Naturally obtained pregnancy after HyFoSy *	73	57
IUI pregnancy after HyFoSy *	14	11
IVF pregnancy after HyFoSy *	41	32

* in the first 3 months after HyFoSy.

**Table 2:** Level of pain during HyFoSy.

Pain Level on Pain Scale	Percentage (%)	Pain Level Compared to Menstrual Pain	Percentage (%)
Absent pain (0)	31	Less painful	54
Tolerable (1–3)	44	Equally painful	15
Painful (4–6)	17	More painful	31
Very painful (7–10)	8		

**Table 3:** Summary of technical tips and tricks and the literature research.

Tips and Tricks We Recommend	Explanation	Literature Data
Scheduling the procedure in the first or second day after the menstrual bleeding cessation	Significantly easier cannulation of the cervix	No data reported
The use of a wedge-shaped pad or of a gynecological table with an easy approach to the sonographic exploration	To ensure a gynecological position associated to the Trendelenburg position as close as possible to 45 degrees; The position favors the gut displacement towards cranial and also the fallopian tubes repositioning as anatomically as possible, so they may be easily spotted	No data reported
The psychological factor—assuring the patient that the procedure is less painful than menstrual pain	Fear causes spasm of the external cervical orifice, which could complicate the cannulation	Fertility can be directly affected by tubal spasm or altered hypothalamic–pituitary pathway due to emotional tensions [28]
The preprocedural preparation with medicinal charcoal and drotaverine hydrochloride	Minimizes gut distention for an optimal visualization of the tubes	Medicinal charcoal is used in reducing bloating [27]
Always test the vaginal flora A single doxycycline dose administration	To prevent infection; There were no consecutive pelvic inflammatory disease or tubo-ovarian abscesses in our study	Prior to certain procedures (HyCoSy, HyFoSy, hysterosalpingography, hysteroscopy, etc.), patients at high risk for pelvic inflammatory disease should be screened and receive treatment [29]
The use of a large size autostatic speculum	To permit maneuvers; The sonographer can use both hands for the cervix cannulation	No data reported
Slowly release of the contrast substance	The substance would be quantitative enough for the visualization of both fallopian tubes	No data reported
Evaluating the uterine cavity at the end of the procedure	The uterine cavity is not distended by the substance; The focal lesions are highlighted by a fine layer of substance	The uterine cavity is evaluated by instilling sterile saline. The uterine cavity evaluation is best performed before instillation of ExEm gel. [30]
Tubal patency evaluation requires evaluating the entire substance passage through the tubes	Visualizing the contrast substance progression through the entire tubal pathway, its evacuation near the ovary, and finding the contrast substance at the end of the procedure in the pouch of Douglas or as a fine hyperechoic line near the uterus	Thin line of contrast substance visualized from the interstitial to the infundibular part of the fallopian tube and contrast substance present in the cul-de-sac are signs of tubal patency [31]
The use of 2D sepia mode	The functional dynamics of the tubes are optimally evaluated; Using 3D mode on HG-Flow offers spectacular images without acquiring a real informational benefit	3D-HyFoSy, with or without Doppler techniques, does not bring additional information compared to 2D-HyFoSy when used by a ultrasonographer who has knowledge of the pelvic anatomy [29]

## Data Availability

The datasets used and/or analyzed during the current study are available from the corresponding author on reasonable request.
